# The Evaluation of Health-Related Quality of Life Issues Experienced by Patients with Desmoid-Type Fibromatosis (The QUALIFIED Study)—A Protocol for an International Cohort Study

**DOI:** 10.3390/cancers13133068

**Published:** 2021-06-22

**Authors:** Anne-Rose W. Schut, Milea J. M. Timbergen, Emma Lidington, Dirk J. Grünhagen, Winette T. A. van der Graaf, Stefan Sleijfer, Winan J. van Houdt, Johannes J. Bonenkamp, Eugenie Younger, Alison Dunlop, Robin L. Jones, Cornelis Verhoef, Spyridon Gennatas, Olga Husson

**Affiliations:** 1 Department of Medical Oncology, Erasmus MC Cancer Institute, 3015 GD Rotterdam, The Netherlands; a.schut@erasmusmc.nl (A.-R.W.S.); m.timbergen@erasmusmc.nl (M.J.M.T.); w.vd.graaf@nki.nl (W.T.A.v.d.G.); s.sleijfer@erasmusmc.nl (S.S.); 2 Department of Surgical Oncology, Erasmus MC Cancer Institute, 3015 GD Rotterdam, The Netherlands; d.grunhagen@erasmusmc.nl (D.J.G.); c.verhoef@erasmusmc.nl (C.V.); 3 Sarcoma Unit, Royal Marsden NHS Foundation Trust, London SW3 6JJ, UK; emma.lidington@rmh.nhs.uk (E.L.); eugenie.younger@rmh.nhs.uk (E.Y.); alison.dunlop@rmh.nhs.uk (A.D.); robin.jones4@nhs.net (R.L.J.); spyridon.gennatas@nhs.net (S.G.); 4 Department of Medical Oncology, Netherlands Cancer Institute, 1066 CX Amsterdam, The Netherlands; 5 Department of Surgical Oncology, Netherlands Cancer Institute, 1066 CX Amsterdam, The Netherlands; w.v.houdt@nki.nl; 6 Department of Surgical Oncology, Radboud University Medical Center, 6525 GA Nijmegen, The Netherlands; han.bonenkamp@radboudumc.nl; 7 Division of Clinical Studies, Institute of Cancer Research, Royal Marsden NHS Foundation Trust, London SM2 5NG, UK

**Keywords:** desmoid-type fibromatosis, rare diseases, health-related quality of life, patient-reported outcomes, disease-specific measures, symptoms, symptom burden

## Abstract

**Simple Summary:**

Desmoid-type fibromatosis (DTF) is an uncommon soft tissue tumour with a high recurrence rate after resection. DTF does not metastasise, but its locally aggressive tumour growth and chronic character can cause significant morbidity. Although overall mortality is low, patients can experience a high symptom burden, making assessment of health-related quality of life (HRQoL) highly relevant. At present, data on HRQoL in DTF patients are limited. The QUALIFIED study aims to (1) pre-test a previously developed DTF-specific HRQoL tool (the DTF-QoL); (2) evaluate prevalence of HRQoL issues in adult DTF patients; and (3) identify subgroups at risk of impaired HRQoL. An international, multicentre, cross-sectional, observational cohort study will be conducted to validate the DTF-specific HRQoL questionnaire and to gain more insight into the issues experienced by DTF patients. This information will help to improve clinical practice, symptom control and patient satisfaction. When validated, the DTF-QoL can be used in both clinical and research settings to evaluate HRQoL of DTF patients.

**Abstract:**

Sporadic desmoid-type fibromatosis (DTF) is a rare soft tissue tumour with an unpredictable clinical course. These tumours are incapable of metastasising, but their local aggressive tumour growth and tendency to recur locally can result in a substantial symptom burden. Measuring the impact of DTF on health-related quality of life (HRQoL) can be challenging due to the variable clinical presentation of the disease. Therefore, a HRQoL instrument assessing DTF-specific issues is needed. The QUALIFIED study aims to (1) pre-test a previously developed DTF-specific HRQoL tool (the DTF-QoL); (2) evaluate prevalence of HRQoL issues in adult DTF patients; and (3) identify subgroups at risk of impaired HRQoL. This study (NCT04289077) is an international, multicentre, cross-sectional, observational cohort study. Patients ≥ 18 years with sporadic DTF from the Netherlands and the United Kingdom will be invited to complete a set of questionnaires specifically composed for this patient group. Questionnaires will be completed using PROFILES (Patient Reported Outcomes Following Initial treatment and Long-term Evaluation of Survivorship). Analyses will include testing the psychometric properties of the DTF-QoL and evaluating the prevalence of HRQoL issues using the DTF-QoL, EORTC QOL-C30 and EQ-5D-5L, among other questionnaires. This study will provide insight into HRQoL issues experienced by patients with DTF. Awareness of these issues and the implementation of the DTF-QoL in research and clinical practice can help to improve overall HRQoL and to provide personalised care.

## 1. Introduction

Desmoid-type fibromatosis (DTF) is an uncommon soft tissue tumour with a highly variable clinical course. Although this tumour does not metastasise, its potential locally aggressive tumour growth can cause significant morbidity. It is therefore classified as an intermediate tumour by the World Health Organisation [1,2]. The biological behaviour of DTF is unpredictable and displays phases of progressive growth or growth stabilisation and up to 28% of tumours ultimately undergo spontaneous regression [3,4,5]. For a substantial proportion of patients, DTF is a chronic condition which should be managed accordingly [6].

In the most recent European consensus guideline, active surveillance is advocated as primary treatment for asymptomatic and mildly symptomatic patients, independent of tumour location or size [7]. Persistent radiological progression or increasing symptoms can necessitate a change from surveillance to active treatment. These treatments include systemic therapies, radiotherapy or (extensive) surgical resection. A recent systematic review reported that approximately one-third of the patients with initial active surveillance approach needed a shift to an active treatment [8]. The type of active treatment mainly depends on anatomical location [7]. Surgery can be considered for abdominal wall tumours as the risk of local recurrence and morbidity is limited [7,9,10]. However, for DTF located at other sites, recurrence rates are high—up to 60%—and unless the risk of morbidity after surgical resection is very low, other therapies, including systemic, are preferred [7,11]. Unfortunately, the evidence for systemic treatments is mainly empirical and randomised controlled trials (RCT) in DTF are limited [12,13]. An RCT comparing sorafenib to placebo reported an overall objective response of 20% in the placebo group vs. 33% in the sorafenib group, with a median time to response of 13.3 and 9.6 months, respectively [12]. Moderate dose radiotherapy has been demonstrated to be effective in patients with inoperable progressive DTF [14]. Radiotherapy can be considered when surgery or systemic therapies are not an option and the risk of serious morbidity is low [2,7,14]. However, because of the relatively young age of the DTF patients, the non-malignant character of the disease and the risk of secondary malignancy after radiotherapy, this modality is not often applied. The main goal of treatment for DTF patients is to maintain acceptable health-related quality of life (HRQoL) by reducing symptoms, obtaining a decrease in tumour size or both. Most importantly, therapeutic management of DTF should never cause more symptoms than DTF itself. Therefore, clinical studies should not only include objective outcomes such as radiological response or overall survival, but also patient-reported outcomes (PROs) [2]. One of the most important PROs is HRQoL, a multidimensional concept that includes the patient’s perception of the impact of their disease and treatment on physical, psychological and social functioning [15]. Several studies have shown that integration of HRQoL assessment in clinical practice can improve patient satisfaction, communication and symptom control, and can guide treatment decision making [16,17]; however, data on HRQoL in DTF patients are scarce.

Up to now, studies have shown that DTF patients experience a variety of physical and psychological symptoms, negatively affecting HRQoL. For example, studies by Husson et al. and Timbergen et al. showed that DTF patients reported physical challenges including pain, treatment complications or side effects and function restrictions [18,19]. Furthermore, DTF patients experienced uncertainties due to diagnostic delay, the unpredictable clinical course, variable treatment efficacies and the risk of recurrence, causing significant distress. Social relationships of some patients were affected due to the uncertainties DTF entails, functional impairment as a result of DTF and/or treatment and the feeling of not wanting to be a burden to others. Some patients mentioned feeling frustrated about their inner circle who had difficulty understanding DTF and the underestimation of the consequences of DTF, since it is categorised as an intermediate tumour. Financial difficulties, due to loss of employment and hospital expenses, were a significant concern [6,18,19]. Additional issues affecting HRQoL include delayed diagnosis and/or misdiagnosis due to the rarity of the disease and the limited experience of healthcare professionals of DTF [18,19,20]. These DTF-specific HRQoL issues are not captured by generic or cancer-generic HRQoL questionnaires (e.g., European Organization for Research and Treatment of Cancer Quality of Life Questionnaire Core 30 (EORTC QLQ-C30) and EuroQol five-dimensional questionnaire (EQ-5D)), which are predominantly used in DTF studies. Only one DTF-specific tool is currently available, the ‘Gounder/DTRF Desmoid Symptom/Impact Scale’ (GODDESS) [21], which is being used in three ongoing clinical trials (NCT04195399 [22], NCT03785964 [23] and NCT03459469 [24]). Although the development of the GODDESS has enhanced our possibilities to assess DTF-specific HRQoL, there are some limitations. The 24 h timeframe for symptom items is short and patient’s answers can be biased by momentary factors. Consequently, symptom items need to be measured multiple times to obtain reliable results. Furthermore, the GODDESS cannot be used in conjunction with cancer-generic questionnaires, such as the EORTC QLQ-C30, to allow comparison with cancer patient populations and normative populations. Finally, the study included patients with familial adenomatous polyposis (FAP)-related DTF. Because of the high incidence of malignant colorectal tumours in FAP patients, these patients might experience different HRQoL issues compared to patients with sporadic DTF. Therefore, we decided to develop a DTF-specific HRQoL questionnaire according to the guidelines of the EORTC Quality of Life Group, that can be used in conjunction with the most widely used cancer-generic HRQoL questionnaire, the EORTC QLQ-C30 [25]. First, we organised focus groups and semi-structured interviews in the United Kingdom (UK) and in the Netherlands [18,19]. The issues identified covered various domains including the diagnostic pathway, the treatment pathway, daily limitations (e.g., physical and psychological symptoms) and experiences with the current healthcare system. Next, the 124 issues identified were ranked according to their relevance by patients and healthcare providers. [6]. Based on the results of these studies, the most relevant issues (102) were operationalised into questions for a DTF-specific HRQoL tool, named the DTF-QoL (phase II EORTC guidelines). Within the QUALIFIED (the evaluation of health-related quality of life issues experienced by patients with desmoid-type fibromatosis) study we will pre-test the psychometric properties of the questionnaire. In addition, the QUALIFIED study aims to evaluate the prevalence of HRQoL issues experienced by adult DTF patients and to explore patient preferences in the decision-making process and healthcare use. This manuscript describes the study protocol for the QUALIFIED study.

## 2. Methods and Analysis

### 2.1. Study Design and Setting

An international, multicentre, cross-sectional, observational cohort study among patients with sporadic DTF treated in one of the participating centres (one centre in the UK: the Royal Marsden Hospital London; three centres in the Netherlands: Erasmus Medical Centre (MC) Rotterdam, Radboudumc Nijmegen, Netherlands Cancer Institute Amsterdam) will be conducted (registered at clinicaltrials.gov: NCT04289077). Patients will be invited to complete a set of questionnaires specifically composed for the patient. After informed consent, patients will be able to complete the questionnaires. Sociodemographic and clinical data will be extracted from medical patients records and the questionnaire (patient-reported). This study was approved by the Royal Marsden and Institute of Clinical Research Joint Committee for Clinical Research for ethical review (SE806) in the United Kingdom, and in the Netherlands, the Institutional Review Board (or Ethics Committee) at each participating centre (Erasmus MC: MEC-2019-0816, Radboudumc: file number 2020-6235, Netherlands Cancer Institute: IRBd20-088). The study started recruitment in August 2020.

### 2.2. Patient and Public Involvement

The QUALIFIED study was designed in collaboration with experts from sarcoma centres in the Netherlands and the UK. The Dutch patient advocacy group (Contactgroep Desmoïd) and Dutch (n = 2) and British (n = 2) patients were asked to comment on the questionnaire. Their comments were used for further adaptation of the wording and the order of the questions. Based on their answers, none of the items were excluded. Local patient advocacy groups will be involved in sharing the study results with patients.

### 2.3. Eligibility Criteria

All adult (≥18 years) patients diagnosed between January 1990 and July 2020, with pathologically proven, sporadic DTF and a recent (between October 2014 and July 2020) visit to the hospital for their DTF are eligible for inclusion in the study. Patients must be able to understand and provide written informed consent to complete a questionnaire. Patients diagnosed with familial adenomatous polyposis (FAP)-related DTF are excluded from the current study because of the different aetiology and treatments which can lead to different HRQoL issues. The inclusion criteria are depicted in Table 1.

### 2.4. Data Collection

PROFILES (Patient Reported Outcomes Following Initial treatment and Long-term Evaluation of Survivorship) will be used for data collection and online questionnaire administration. PROFILES is a data management system set up in 2009 in the Netherlands for the study of the physical and psychosocial impact of cancer and its treatment [26]. The PROFILES management system is stored on a secured server in the Netherlands in accordance with current European norms (NEN-ISO/IEC 27002). A separate version of PROFILES was recently installed in the Royal Marsden Hospital (London, UK) to meet local information governance requirements. Data from patients in each country will be collected and stored in the local PROFILES system.

### 2.5. Recruitment

Patients will be selected (based on their diagnosis) by the responsible treating consultant who will check the patient for their eligibility using information from the electronic patient records. The treating healthcare professional will introduce the study to the patient in the outpatient clinic and provide an invitation package. In case the patient does not visit the outpatient clinic within two months of the start of the study, the treating healthcare professional will invite the patient with the invitation package by post. Patients will be assured that non-participation has no consequences for their treatment or follow-up care.

The invitation package consists of a letter, a patient information sheet, an informed consent form and a prepaid return envelope. The patient information sheet explains the goals and procedures of the study and includes a link and login codes to the secure PROFILES website. Interested patients will be given the option to participate online or using a paper version of the questionnaire. Those who prefer online participation, and who have read the information sheet without further questions, can login to PROFILES with their unique login name and password. If the patient does not have access to the internet or a computer, or prefers written rather than digital communication, they can request a paper version of the questionnaire package. Paper copies of the questionnaires will be entered by the research coordinator using the data entry option of PROFILES.

Informed consent will be obtained from all patients. This can be done on paper if a patient prefers a paper version of the questionnaire. Those who prefer online participation will be able to complete the questionnaire online after providing both written and online informed consent.

Patients will fill out the questionnaire only once. Completing all the questionnaires will take about 30–60 min. The link to the online questionnaire will be available in PROFILES for a total of three months after invitation. Patients are able to stop and start at any given time point and save their answers. In case the questionnaire is only partially completed, the patient will receive a reminder (via PROFILES) to fill out the remaining items. All patients will receive a reminder four weeks after the first invitation.

### 2.6. Case Report Forms

Sociodemographic and clinical data will be collected in the questionnaire (patient-reported) and from the medical files of patients who provided informed consent. Clinical data collected from the medical records include details regarding the pathology, treatment strategies and recurrence, including treatment start date, type of treatment(s), medication dose(s), radiation dose(s), date(s) of surgery, sequence of treatments, response to treatments, date(s) of recurrence(s) and treatment type(s) for recurrence(s). The information will be maintained according to ICH-GCP (international good clinical practice) standards and entered into the study’s password-protected database where it will be stored on case report forms (CRF) using non-identifiable study numbers. The patient records will not leave the hospital where the patient is treated. The questionnaire data will be linked with the clinical data with the CRF database using patient study numbers. Data will be linked by an epidemiologist at the Erasmus MC. Data will be stored in accordance with the Dutch General Data Protection Regulation.

### 2.7. Questionnaires

Newly created questions and existing validated questionnaires were combined for this study. The total questionnaire package contains 173 questions.

#### 2.7.1. Sociodemographic and Clinical Characteristics

The sociodemographic and clinical characteristics questionnaire includes single items on age, sex, race, marital status, family composition, educational level, working situation, comorbidities, tumour location, details regarding the diagnosis, received treatments, recurrent tumours and the current treatment strategy. Health literacy will be assessed by one single item question [27]. Additional medical data will be obtained from the electronic patient records to ensure correct and detailed reporting. If the patient-reported clinical data are inconsistent with the data from the electronic patient record, the latter will be used for statistical analysis.

#### 2.7.2. Healthcare Utilisation

The ‘healthcare utilisation’ questionnaire is included to gain more insight into the use of medical facilities by DTF patients and their satisfaction with the care received. Patients will be asked about the frequency and reasons (DTF-related or not) for contact with their general practitioner or medical specialist in the past twelve months. Furthermore, patients will be asked about their preferences for follow-up and whether they have been referred to other healthcare services (e.g., psychologist, physical therapist, etc.) [28,29,30].

#### 2.7.3. Decision Making

Six questions have been adapted by our group to provide information on how patients make medical decisions, what their current role is and what their preferred role in the decision-making process is [31]. These questions were taken from the ‘Control Preference Scale’ and the ‘Decisional Conflict Scale’ and adjusted to the context of DTF [31,32]. Roles are classified as fully active, active–collaborative, collaborative, passive–collaborative or fully passive [32]. The four-item Decisional Conflict Scale (‘SURE’) was used to gain insight into the awareness of the benefits and the risks of certain treatments. This includes the four items; ‘Sure of myself’, ‘Understand information’, ‘Risk-benefit ratio’ and ‘Encouragement’, with two answer categories: yes (score 1) or no (score 0). The ‘SURE’ Decisional Conflict Scale Questionnaire has been validated in English and a Dutch translation is available (psychometric properties have been partly confirmed in Dutch patients) [33]. Furthermore, two questions were designed to gain insight into the reasons for choosing an active form of treatment.

#### 2.7.4. EORTC QLQ-C30

HRQoL will be assessed with the European Organization for Research and Treatment for Cancer Quality of Life Questionnaire (EORTC QLQ)-C30, version 3.0 [34]. This 30-item HRQoL questionnaire consists of five functional scales (physical, role, cognitive, emotional and social), a global quality of life scale, three symptom scales (fatigue, pain, nausea and vomiting) and a number of single items assessing common symptoms (dyspnoea, loss of appetite, sleep disturbance, constipation and diarrhoea) and perceived financial impact of the disease. A higher score on the functional scales and global quality of life means better functioning and HRQoL, whereas a higher score on the symptom scales means higher symptom burden.

#### 2.7.5. DTF-QoL

The DTF-specific questionnaire, the ‘DTF-QoL’, was designed based on previous focus groups, patient interviews and by ranking the relevance and importance of the issues [18,19]. Although this questionnaire is not an EORTC product, their guidelines for ‘developing a questionnaire’ were used, except for the number of countries in phase I–III as we only included a Northern-European and English-speaking country [25]. Where possible, existing items from the EORTC-item library were used and duplicate items from the EORTC QLQ-C30 were removed. This resulted in a 102-item questionnaire (DTF-QoL) describing DTF-specific issues. Patients will be asked to score each item on a Likert scale ranging from 1 to 4 ((1) not at all, (2) a little, (3) quite a bit, and (4) very much). Questions will be asked in different time frames in order to minimise failure to recall but also to maximise the ability of patients to respond, as patients with various phases of disease are included in the current study [35]. Eighteen questions have the time frame ‘during the last week’. One question has the time frame ‘in the last four weeks’. The remaining questions have the timeframe ‘since your diagnosis’. Covered topics include psychosocial, physical, diagnostic and treatment domains.

#### 2.7.6. EQ-5D-5L

The EuroQol five-dimensional questionnaire (EQ-5D-5L) is a descriptive system for the measurement of health. It measures quality of life (QoL) in five dimensions: mobility, self-care, usual activities, pain–discomfort and anxiety/depression [36]. Each of the five dimensions of the EQ-5D-5L can be divided into five levels of perceived problems ((1) no problems, (2) slight problems, (3) moderate problems, (4) severe problems and (5) extreme problems). The response value of each domain is combined into a 5-digit value that indicates a corresponding health state. Health states can be converted into single index values using country-specific reference data. A vertical visual analogue scale (VAS), the EQ VAS, records the patient’s self-rated health. Patients can give a score from 1 to 10 with the endpoints labelled as ‘The best health you can imagine’ (10) and ‘The worst health you can imagine’ (1).

### 2.8. Objectives

The primary objective of the study is to (1) pre-test the psychometric properties of a previously developed DTF-specific HRQoL tool according to the phase III EORTC guidelines. The secondary objectives are: (2) to evaluate the prevalence of HRQoL issues in adult DTF patients, regardless of their disease phase or treatment course, (3) to compare the scores of the DTF patients to those of the general population (matched to age and sex and based on the scores of the EORTC QLQ-C30), (4) to identify patients at risk of impaired HRQoL (based on the analysis of subgroups using the EORTC QLQ-C30 and the DTF-QoL), (5) to evaluate the treatment decision-making process and preferences and (6) to evaluate the healthcare use of DTF patients.

### 2.9. Sample Size

A sample size calculation is challenging due to the rarity of the disease, the lack of published data regarding this subject and the fact that the diagnosis of DTF is neither part of a cancer registry nor has a disease-specific registry in the UK or the Netherlands. The incidence of DTF is 5.4 patients per million per year in the Netherlands [37]. In our previous Dutch study, the response rate of the Dutch patients was 42% [6]. A small fraction of the remaining patients will be excluded as they are younger than 18 years (about 5%) or their desmoid tumour is part of the genetic syndrome FAP (about 5%). With the participation of three Dutch centres, we hope to approach a total of 250 patients in the Netherlands. Based on the previous response rate, we expect to obtain data from 100 DTF patients. There are no studies describing the incidence of DTF in the UK. All eligible DTF patients were identified from the hospital database of the Royal Marsden Hospital. In the previous study, the response rate of the UK patients was 37.5% [6]. Therefore, we aim to include 70 patients from the Royal Marsden Hospital, London, UK.

### 2.10. Statistical Analysis

The study population will be described using descriptive statistics (mean, SD, median, range, frequencies).

Pre-testing the psychometric properties of the DTF-QoL (study aim 1) will be performed in agreement with the methodology of the phase III EORTC guidelines [25]. Item descriptive statistics and response distributions for each item will be calculated, in order to examine central tendency, variability and symmetry. To support construct validity, factor analysis will be used to determine underlying constructs, which explain significant portions of the variance. The factor loadings will be examined in order to explain the meaning of each construct. Scores of the founded scales will be calculated according to the guidelines of the EORTC quality of life group [38]. Multi-trait scaling analysis will be performed to confirm the hypothesised scale structure of the questionnaire [39]. To test for item–scale convergent validity, the correlation between each individual item and its own scale (corrected for overlap) will be examined. Correlations of ≥0.40 will be considered substantial and satisfactory. By comparing the correlation of each item with its own scale versus other scales, item–scale discriminant validity will be examined. It is expected that an item will show a greater correlation with its hypothesised scale compared to other scales. Reliability will be assessed by calculating Cronbach’s alpha coefficient (ranges between 0 and 1). A minimum score of 0.70 is preferred [40]. Substantially lower scores indicate an unreliable scale. Finally, Pearson’s correlation coefficients will be calculated between the scales of the EORTC QLQ-C30 and found scales of the DTF-QOL to assess convergent and divergent validity. Scales conceptually related are expected to show high correlations (r ≥ 0.40), while scales with less conceptual relation are expected to correlate weakly (r < 0.40).

The prevalence of HRQoL issues (study aim 2) will be evaluated using the scores of the EORTC QLQ-C30 (version 3.0), DTF-QoL and EQ-5D-5L. The scores of the EORTC QLQ-C30 will be calculated using a Likert scale from 1–4. The scoring manual of the EORTC will be followed [38]. After linear transformation, all scales and single-item measures will range in score from 0 to 100. Scores for each scale will be reported as mean (SD), or as median (interquartile range (IQR)).

The scores for each item of the DTF-QoL will be calculated using a Likert scale from 1–4. The scores of each item will be calculated as mean ± standard deviation (SD), or as median (IQR). These scores (median or mean) will be reported per item. Scores of the developed scales of the DTF-QoL will be calculated according to the guidelines of the EORTC quality of life group [38].

Each of the five dimensions of the EQ-5D-5L (version 1.0) can be divided into five levels of perceived problems (1–5). Health states can be converted into single index values. The outcomes will be reported as frequency (proportion) of reported problems for each level and for each dimension. The VAS data will be presented as a mean value (SD). In case of skewed data, median values and IQR will be used.

To compare the scores of DTF patients with the general population (study aim 3), the demographic and clinical data for age and sex will be used and patients will be matched, using a 1:10 nearest-neighbour match method, with the general population [41] based on age and sex, using RStudio (RStudio, version 1.0.153, Boston, MA, USA, package MatchIt). Differences in scores of the EORTC QLQ-C30 scales between groups will be tested for their significance using the Mann–Whitney U test or the *t*-test, depending on the distribution.

Multiple linear regression analyses will be conducted to investigate the independent associations between clinical and sociodemographic characteristics with subscale scores of the EORTC QLQ-C30 and DTF-QoL (study aim 4). Subgroups will be created based on demographic and clinical information.

The outcomes of the healthcare utilisation (study aim 5) and the decision-making questionnaire (study aim 6) will be reported in numbers and corresponding percentages per answer option. The total score of the ‘SURE’ Decision Conflict Scale will be calculated if all four items are answered. The sum of the four items will range from 0 (extremely high decisional conflict) to 4 (no decisional conflict). A score of ≤ 3 indicates decisional conflict [31].

All statistical analyses will be performed using SPSS Statistics (IBM, Armonk, New York, NY, USA, version 25.0). A two-sided *p* < 0.05 will be considered statistically significant.

Statistical techniques to deal with imbalanced data due to the rare character of DTF will be considered (e.g., artificial intelligence techniques).

### 2.11. Missing Data

Participants who have completed online questionnaires will not have missing data, unless they did not complete the entire questionnaire. The online questionnaires have been programmed so that participants are unable to proceed to the next question until all questions on the current page have been answered. The number of items missing from paper questionnaires will be described where applicable, and only available data will be analysed. For missing data of the EORTC QLQ-C30, the EORTC scoring manual guideline will be followed [38].

## 3. Conclusions

The unpredictable and highly variable clinical course of DTF causes a variety of HRQoL issues in DTF patients, indicating the need to assess the impact of DTF on patients’ lives. This international, cross-sectional, observational study will pre-test a previously developed DTF-specific HRQoL tool (the DTF-QoL); provide insight into HRQoL issues experienced by DTF patients; compare the HRQoL of DTF patients with the ‘general population’; and identify patients at risk for a poor QoL. When validated, the DTF-specific HRQoL tool, accompanied by the EORTC QLQ-C30, could be a useful HRQoL instrument in future (longitudinal) clinical studies and clinical care. Furthermore, it could help healthcare professionals to recognise HRQoL issues earlier and thereby improve overall patient experience and overall quality of life.

## Figures and Tables

**Table 1:** Inclusion and exclusion criteria for participation in the QUALIFIED study.

Inclusion Criteria	Exclusion Criteria
Patients aged ≥ 18 years	Patients diagnosed with FAP-related DTF
Patients with histopathological proven DTF, regardless of disease phase or treatment.	
Patients diagnosed between January 1990 and July 2020, with a visit to the hospital for their DTF (between October 2014 and July 2020)	
Patients with sufficient Dutch/English language skills	
Patients competent to complete a questionnaire	
Patients with written informed consent	

DTF, desmoid-type fibromatosis; FAP, familial adenomatous polyposis.

## Data Availability

Data sharing is not applicable.
